# “Metal-modified base pairs” vs. “metal-mediated pairs of bases”: not just a semantic issue!

**DOI:** 10.1007/s00775-022-01926-7

**Published:** 2022-01-29

**Authors:** Bernhard Lippert

**Affiliations:** Fakultät Für Chemie Und Chemische Biologie (CCB), Technische Universität Dortmund, 44221 Dortmund, Germany

**Keywords:** Metal-modified nucleobase pairs, Metal-mediated base pairs, Metal nucleobase cross-links

## Abstract

A “nucleobase pair” is not identical with a “pair of basic ligands”, as only in the first case, the existence of inter-base hydrogen bonds is implied. The cross-linking of two nucleobases or two basic ligands by a metal ion of suitable geometry produces either “metal-modified” or “metal-mediated” species, but in the author’s opinion, this difference is not always properly made. This commentary is an attempt to provide a clearer distinction between the two scenarios.

**Graphical abstract:**

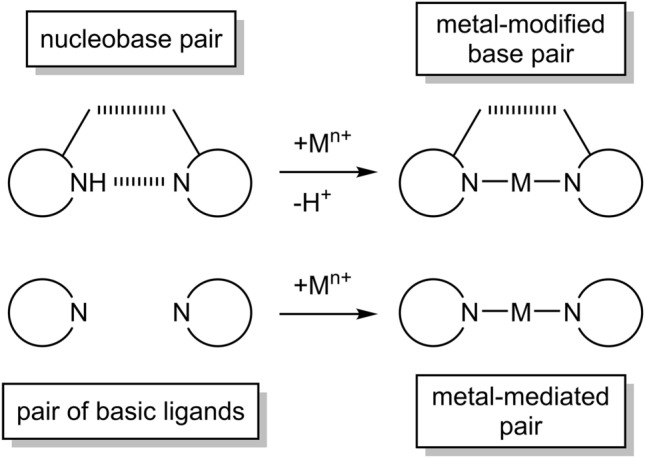

The past few decades have witnessed a plethora of structural and spectroscopic reports on metal ions cross-linking natural nucleobases, model nucleobases, or artificial nucleobase surrogates [1–3]. The potential relevance of such “metallo-base pairs” and their applications ranges from biology (e.g., tautomerization of nucleobases [4]) to analytical chemistry (e.g., DNA-based metal sensors [5]), supramolecular chemistry (e.g., metallacyclic compounds [6]), materials chemistry (e.g., nanowires [7], DNA hydrogels [8], fluorescent metal clusters [9]), and medicinal chemistry (e.g. antisense or antigene approaches [10]), among others [11].

The term “metal-modified base pair” has originally been used by ourselves for describing cross-linking adducts of (mostly) linear metal entities with canonical natural nucleobases, in which hydrogen bonds are lost at the expense of coordinative metal–ligand bonds, usually associated with the release of a proton from the original H bond [12–14]. Lately, the term “metal-mediated base pairs” has come into more frequent use. Occasionally, both definitions are being used synonymously. While both terms are justified at their own right (see below), a more precise differentiation is desirable. Herewith, the following distinction is proposed:

A base pair in its original meaning is an essentially planar entity comprising two natural nucleobases, held together by hydrogen bonds. Apart from the classical Watson–Crick and Hoogsteen hydrogen-bonding patterns between the complementary purine (guanine, G, adenine, A) and pyrimidine nucleobases (cytosine, C, thymine, T, uracil, U), a large number of alternative pairing patterns have been discovered over the years, which include hydrogen-bonded mispairs between non-complementary bases, self-pairs, pairs involving protonated or deprotonated bases, pairs containing rare nucleobase tautomers, solvent (H_2_O)-containing pairs, as well as pairs which in addition to H bonds also use alkali metal ions for stabilization [15]. Starting from there, any replacement of a hydrogen bond by a metal ion cross-link represents a modification of the original pair according to the notion that a “modification is a (minor) change in an existing entity”. The resulting metallo-base pair should thus be termed a “metal-modified base pair”. It may or may not keep any of its original additional H bonds. Minor alterations of canonical nucleobases not affecting base pairing properties greatly (e.g., small substituents at the heterocyclic rings, deaza forms, *iso* forms of nucleobases such as iC or iG) should be included in these considerations. Representative examples are compiled in refs. [16–21]. Depending upon the metal ion (or metal entity, if co-ligands are present), its preference for N and/or O donor sites, and its preferred coordination geometry (linear, trans-square planar, trigonal planar, tetrahedral), different types of modified base pairs are feasible. For example, Hg^II^ cross-linking of a TT mispair (with loss of protons) can occur between two N3 sites, between N3 of one T and O4 of the other T, or between N3 of one T and O2 of the other. All three options in fact occur in an antiparallel DNA duplex (sugar entities in cisoid orientations), albeit with different frequencies [20, 21] (Fig. 1). Depending upon pH, any of these “metal-modified TT base pairs” could retain one of its two acidic protons, thereby stabilizing the cross-link and forming a metallo-base pair containing a T anion and a neutral T, or a T anion and a rare T* tautomer, respectively. In reverse, starting from a feasible TT* mispair [22], any of these cross-links could be generated. Observations that they can bind additional metal ions [19, 21] instead of a proton have previously been demonstrated in model complexes [23].

**Fig. 1:**
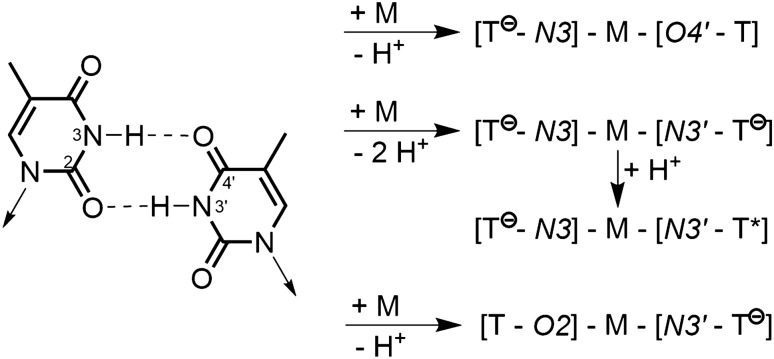
TT wobble pair (left) and variants of metal-modified pairs. Metallopairs containing the metal coordinated exclusively through O-donors are not considered. *T* neutral thymine, *T*^*−*^ thymine anion, T* rare 4-hydroxo,2-oxo tautomer of thymine

As to the role of co-ligands at the metal, if not too bulky, they do not necessarily have a large negative effect on a helix structure. Thus, the two NH_3_ ligands of *trans-*(NH_3_)_2_Pt^II^ present in the DNA interstrand cross-link between G-*N7* and C-*N3*, formally derived from the Hoogsteen pair between protonated C and G, have little influence on the overall structure [24]. With model cross-links, this steric aspect is irrelevant anyway.

We propose to use the term “metal-mediated surrogate base pair” exclusively for any metal cross-links of ligands (including two different ones) present in oligonucleotide structures, that are not natural nucleobases or close variants of these. In general, “metal mediation” of two basic ligands by a metal ion does not require release of a proton, nor the loss of a hydrogen bond. With most of the published ligands applied in these pairs, this statement is unambiguous [25–30]. With Müller’s azole ligands, and particular with the *N1-*imidazole one (p*K*_*a*_ ca. 6.5), this assumption may be challenged, as a hemiprotonated species for N-substituted imidazole in physiological pH seems reasonable, yet such an associate has not been experimentally confirmed [28] (Fig. 2).

**Fig. 2:**
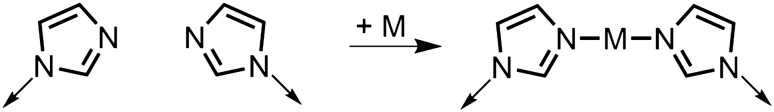
Metal-mediated pair of two imidazole ligands

Consequently it may be asked whether the term “base pair” indeed is appropriate at all in this context, or whether these entities rather represent “pairs of basic ligands”, connected by a metal ion, with “basic” referring to their property to provide lone electron pairs.

Unfortunately, the above used differentiation between “modification” and “mediation” is not always clear-cut, not even for natural nucleobases (or slight variations of these). Rather, it depends on the point of view, and there are cases of ambiguity. For example, a metal cross-link between two G-*N7* sites may be seen as a typical case of “metal-mediation”, as there is no H bond between the donor sites of two neutral Gs [7]. However, if such a metallo-pair is conceptually derived from the existing hemiprotonated [GHG]^+^ pair [31], its proper description would then be a “metal-modified base pair”. Cross-links between two N1 positions or combinations of N1/O6 or N1/N2 represent clear cases of “modifications”. On the other hand, metal cross-links between two N7 sites of A, two N1 sites of A, or a combination of N1 and N7, can be seen as “mediated pairs”, unless the existence of a rare tautomer structure of one of the two As or of a protonated A is evoked. At least the existence of AH^+^ in nucleobase pairs (with C and G) is well established [15]. Another case of ambiguity!

Moreover, the nature of the cross-linking metal ion and its coordination geometry eventually may decide, which definition is more appropriate. For example, Shionoya’s 5-carboxyuracil (caU) ligand [32] forms a mixed pair with T in the presence of Hg^II^, and a mispair with C and Ag^I^, both of which are to be considered clear cases of “metal-modification” according to our definition. The homo pair with a Cu^II^ cross-link and likewise the mixed Cu^II^-caU/G metallo-pair are more ambiguous in this respect, as structural information concerning the pairing scheme between two caU molecules as well as between caU and G in the absence of metal ions is not available. Consequently, the term “metal-mediated” pairs, as used by the authors, is justified. However, at least the Cu^II^(caU)_2_ cross-link could, in principle, be derived from a hypothetical, caU pair (with hydrogen bonds between O2/N3H of one entity and O4/N3H of the other one), followed by twofold metal chelation, the loss of two hydrogen bonds, yet no loss of protons. If real, the metal adduct would then represent a “metal-modified” pair. To evoke a pairing scheme between caU and G via the Hoogsteen edge of G, and hence a “Cu-modification” of such a base pair, would require additional prerequisites (tautomerization; inclusion of water), which we do not wish to further speculate on.

Special attention must be given to the numerous studies relating to Ag^I^-cross-links of CC. Two situations need to be differentiated: first, when the two Cs have their glycosidic bonds mutually cisoid, as is the case in an antiparallel duplex, one might argue that the two Cs do not form any H bonds and hence a metal cross-link leads to a “metal-mediated pair”. However, if an asymmetrical, water-mediated CC pair is taken as a starting point, as observed in an RNA duplex [33], CC indeed is a “base pair” according to our definition, and Ag^I^ cross-linking via the two N3 sites leads to a complete loss of the original five H bonds, even though not to release of a proton. Hence, in our view, the proper term would be “metal-modified CC pair”. The same applies, if one starts out from the established mismatch seen in [CHC]^+^ (not identical with the i-motif to be discussed below!), hence the mismatch representing a hydrogen-bonded “Watson–Crick”pair [15]. All four feasible metal cross-links, and certainly also the cross-link between the two N3 positions, represent “metal-modified pairs” of two neutral C nucleobases (Fig. 3). It must be noted that in some of these cross-links, one of the Cs could adopt a rare tautomeric form (C*). That such scenarios are indeed feasible has unambiguously been demonstrated by us in two X-ray crystal structures containing linear *trans-*X_2_Pt^II^ entities (X=Cl and I) and the preferred aminooxo and the rare iminooxo tautomer of C bonded simultaneously to the metal [34].

**Fig. 3:**
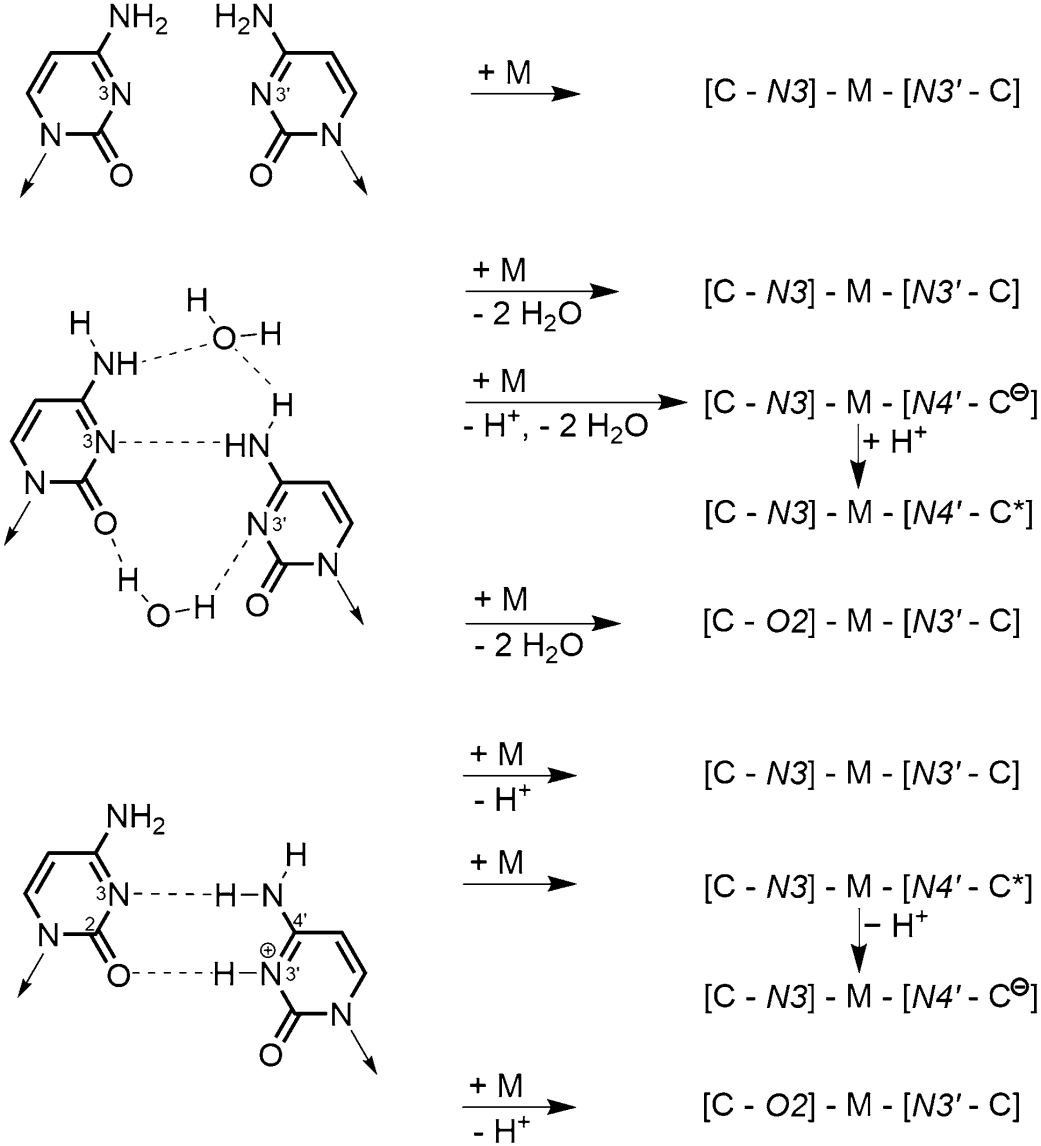
“Metal-mediation” of hypothetical CC pair (top) and variants of “metal-modifications” of established CC and CHC^+^ mispairs possible in antiparallel double helices. *C* neutral cytosine, *C*^*−*^ cytosine anion (deprotonated at N4 position), C* rare iminooxo tautomer of cytosine

Second, the two Cs have their glycosidic bonds arranged transoid, as is possible in a parallel-stranded duplex. This situation is realized in the so-called “i-motif” of hemiprotonated C, hence the [CHC]^+^ pair, with its three hydrogen bonds. Altogether five versions of “metal-modified base pairs” can be derived from this structure, including the cross-link involving the two N3 sites (Fig. 4), which certainly is the most stable one [9]. Clearly, the term “metal-modified CC pair” is appropriate. Given the relatively large tilt angles between C nucleobases in relevant model compounds and their intra- rather than inter-base H bonds [35, 36], this aspect nevertheless calls our interpretation into question.

**Fig. 4:**
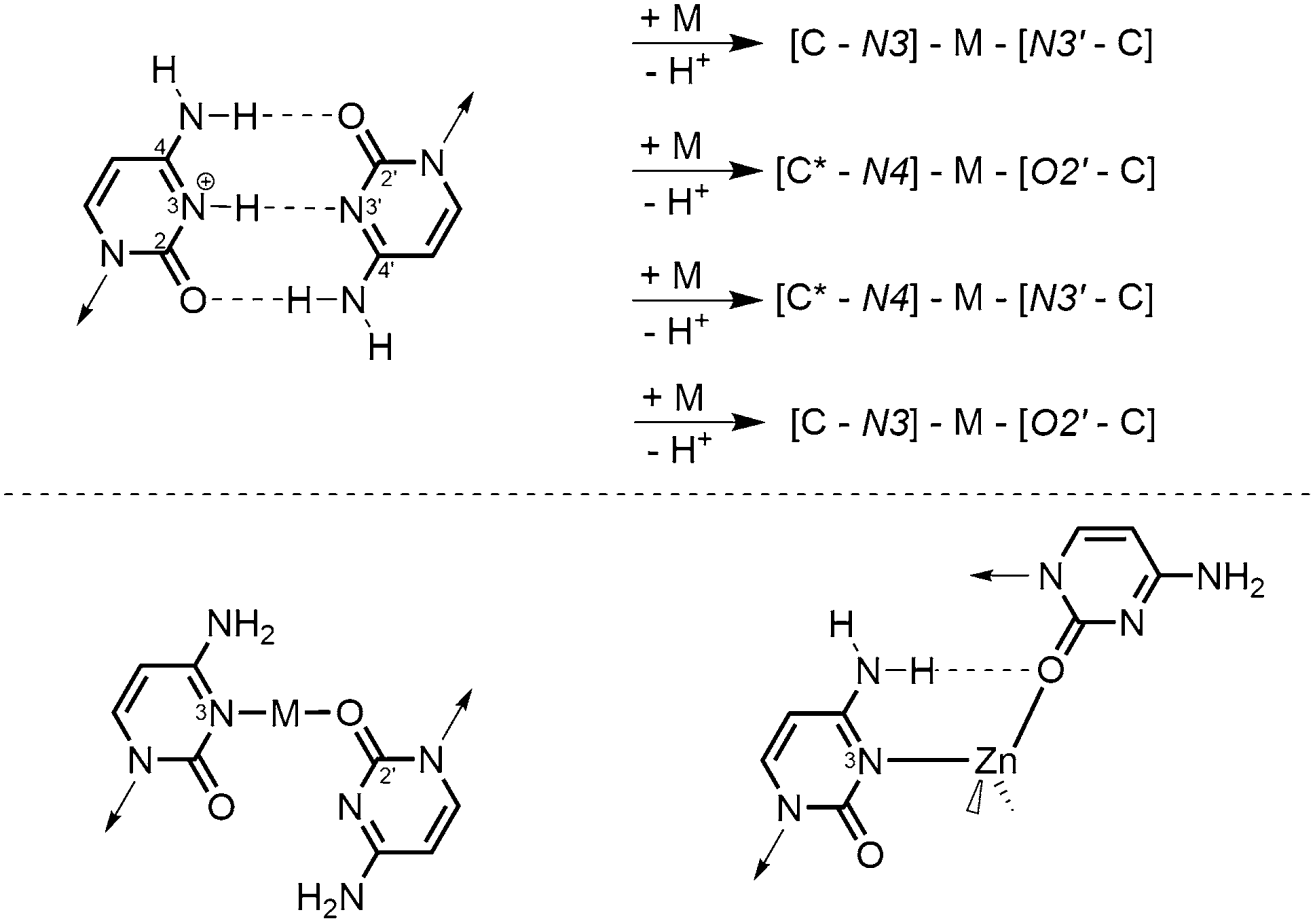
CHC^+^ pair (“i-motif”) possible in parallel-stranded helices and feasible metal-modified versions (top). A variant of [C-*N3*]-M-[*O2′*-C] containing tetrahedral Zn^2+^ instead of a linear M is given below [37]

A variant of one of these patterns, involving N3 and O2 sites, is also realized in a complex containing tetrahedral Zn^II^ and two 1-methylcytosine model bases [37]. The difference between the situation in a linearly cross-linked entity and one containing a tetrahedral metal ion is that, as a consequence of the angle at the Zn ion, the glycosidic bond is substantially rotated as compared to cross-linking by a metal ion of linear coordination geometry. Proposals by Lee et al. on Zn^II^ cross-links of nucleobases in duplex DNA, which are associated with proton loss, are in line with our suggestion [38].

In conclusion: Having analyzed a large series of metallo-base pairs between complementary nucleobases as well as self- and mispairs (not discussed here in further detail), it is felt that, with very few exceptions, these eventually can be traced back to real “nucleobase pairs”, and, consequently, should be termed “metal-modified base pairs”. In contrast, metal cross-links between pairs of basic ligands not identical with common natural nucleobases should be named “metal-mediated pairs” or, when present in nucleic acid structures, “metal-mediated surrogate base pairs”. We are fully aware that the above differentiation becomes increasingly difficult if strongly altered nucleobases (e.g., pyrollo derivatives or “hyper-modified” RNA bases) are taken into account.
